# Health-related quality of life after definitive chemoradiotherapy in patients with esophageal carcinoma: a population-based analysis

**DOI:** 10.1007/s00520-026-10450-2

**Published:** 2026-03-10

**Authors:** D. Wekking, F. H. Erdmann, L. M. Veen, S. C. Kuijper, M. Pape, P. S. N. van Rossum, P. M. Jeene, K. J. Neelis, M. Slingerland, O. Loosveld, T. Rozema, M. A. G. Sprangers, R. H. A. Verhoeven, H. W. M. van Laarhoven

**Affiliations:** 1 Department of Medical Oncology, Amsterdam UMC Location University of Amsterdam, Amsterdam, the Netherlands; 2 Cancer Treatment and Quality of Life, Cancer Center Amsterdam, Amsterdam, the Netherlands; 3 Department of Research & Development, Comprehensive Cancer Organisation (IKNL), Utrecht, the Netherlands; 4 Department of Radiation Oncology, Amsterdam UMC Location University of Amsterdam, Amsterdam, the Netherlands; 5 Radiotherapiegroep, Deventer, the Netherlands; 6 Department of Radiation Oncology, Leiden University Medical Center, Leiden, the Netherlands; 7 Department of Medical Oncology, Leiden University Medical Center, Leiden, the Netherlands; 8 Department of Medical Oncology, Amphia Hospital, Breda, the Netherlands; 9 Verbeeten Institute, Breda, the Netherlands; 10 Department of Medical Psychology, Amsterdam UMC Location University of Amsterdam, Amsterdam, the Netherlands

**Keywords:** Esophageal cancer, Definitive chemoradiation, Health-related quality of life, Patient-reported outcome, Long-term follow-up

## Abstract

**Purpose:**

Definitive chemoradiotherapy (dCRT) can achieve durable local control and even cure in patients with locally advanced esophageal cancer. However, survival benefit may be accompanied by a decline in health-related quality of life (HRQoL) owing to experienced adverse effects. This study aims to investigate HRQoL in patients with locally advanced esophageal cancer receiving dCRT in a real-world setting.

**Methods:**

Patients with locally advanced esophageal squamous cell carcinoma or adenocarcinoma receiving dCRT (≥ 50.4 Gy/28 fractions with concomitant weekly chemotherapy) were eligible. Patient-reported outcome measures were prospectively collected using the validated questionnaires EORTC-QLQ-C30 and EORTC-QLQ-OG25 at baseline and every 3 months thereafter for 2 years. Clinical data were obtained from the Netherlands Cancer Registry. Longitudinal HRQoL outcomes were compared to baseline using mixed effect models.

**Results:**

318 patients were included with a median age of 70 years. The total number of available questionnaires across all timepoints was 718. Out of 318 alive patients, 223 (70.1%) returned a questionnaire at baseline, which declined to 71 out of 195 (36.4%) at 2 years. Global health status at baseline was 70.6 (95% CI 68.1–73.1) and remained stable over time. Patients reported significantly lower physical (-13.0), role (-18.9), cognitive (-6.6), and social functioning (-14.4) at 3 months compared to baseline (all *p* < 0.0001). Social and cognitive functioning scores recovered to baseline level at 6 months. Physical and role functioning, and the symptom scales fatigue and dyspnea, remained impaired until 2 years after baseline. Significant improvements were observed for anxiety, eating restrictions, odynophagia and dysphagia at nearly all time points, but most pronounced at 12 or 18 months.

**Conclusions:**

Our study showed that where global health status remained stable from 6 months after dCRT, the burden of disease-specific symptoms decreased. Social and cognitive functioning first deteriorated but recovered over time, whereas the decline in physical functioning, role functioning, fatigue and dyspnea did not recover to baseline level. These findings can provide valuable insights to address concerns regarding the impact of dCRT on HRQoL within the context of shared decision-making.

**Supplementary information:**

The online version contains supplementary material available at 10.1007/s00520-026-10450-2.

## Introduction

Esophageal carcinoma (EC) is the sixth leading cause of cancer death worldwide with 5-year survival rates ranging from 15 to 25% [1, 2]. In Europe, a standard of care for resectable locally advanced EC is trimodality therapy, consisting of neoadjuvant chemoradiotherapy followed by esophagectomy, and in selected cases, adjuvant nivolumab [3]. Patients with unresectable locally advanced EC, who are unfit for surgery or who opt out of surgery, can be treated with definitive chemoradiotherapy [4]. Since the introduction of definitive chemoradiotherapy, 3-year survival rates for this subgroup have increased from 10% to 28–42% [5, 6]. Across the world, differences can be found in the systemic component of definitive chemoradiotherapy. The most common chemotherapy regimen employed in the Netherlands is carboplatin and paclitaxel, which was established as a result of the CROSS trial demonstrating the efficacy of neoadjuvant chemoradiotherapy [7]. Subsequently, the ARTDECO trial demonstrated efficacy of this regimen in a definitive chemoradiotherapy setting.[5] Other chemotherapy regimens include oxaliplatin and fluorouracil, cisplatin and fluorouracil, and paclitaxel and cisplatin [8, 9].

Regrettably, definitive chemoradiotherapy may be accompanied by late complications, including esophageal stenosis, radiation pneumonitis, and an increased risk of cardiac events [10, 11], which can significantly impact health-related quality of life (HRQoL). Acknowledging the importance of HRQoL in the shared decision-making process, it is worth noting that it has also been demonstrated as a prognostic factor for survival in patients with EC [12]. Previously, a systematic review into HRQoL after definitive chemoradiotherapy, in which the majority of patients received cisplatin-based chemoradiotherapy, reported impaired HRQoL at baseline [13], which improved after treatment. Nevertheless, there is a notable gap in the existing literature regarding information on long term real-world HRQoL following definitive chemoradiotherapy, particularly in cases where carboplatin and paclitaxel is utilized. Therefore, we aimed to conduct a longitudinal examination of the course of HRQoL in patients with locally advanced EC after definitive chemoradiotherapy with carboplatin and paclitaxel in a real-world setting over a period of two years.

## Methods

### Study design and data source

Patients diagnosed with first primary esophageal or gastro-esophageal junction carcinoma with a cT_1-4a_cN_0–3_cM_0_ disease stage were included. Patients with cM_1_ disease based solely on the basis of supraclavicular lymph node metastases were also included, as the prognosis of these patients is similar to cM_0_ patients [14]. Patients were eligible if they received chemoradiotherapy consisting of at least one infusion of carboplatin (area under the curve 2 mg/mL/min) and paclitaxel (50 mg/m^2^) with concurrent radiotherapy (≥ 42.0 Gy or duration of treatment ≥ 30 days to distinguish neoadjuvant and definitive chemoradiotherapy); were diagnosed between 2015 and 2025; were enrolled in the Prospective Observational Cohort study of Oesophageal-gastric cancer Patients (POCOP) and the nationwide Netherlands Cancer Registry (NCR) (see below); and had completed at least 1 HRQoL questionnaire.

Clinical data such as age, sex, and tumor stage were obtained from the NCR. HRQoL outcomes were extracted from the POCOP registry, which is a Dutch observational cohort of more than 4500 patients with esophagogastric cancer [15.] Participants were sent HRQoL questionnaires before start of treatment (baseline), then every 3 months during the first year, and every 6 months during the second year.[15] Questionnaires were considered completed if they were dated within prespecified windows for baseline (before start of treatment or within 7 days after start of treatment); time points 3, 6, 9 and 12 months (± 4 weeks); and time points 18 and 24 months (± 8 weeks). All patients provided written informed consent for participation in POCOP and for linkage with the NCR. According to the Central Committee on Research involving Human Subjects, this type of study does not require approval from an ethics committee in the Netherlands.

### Questionnaires

This study included the European Organisation of Research and Treatment of Cancer (EORTC) Quality of Life Questionnaire (QLQ)-C30 and QLQ-OG25 available through POCOP. The QLQ-C30 (v3.0) is a validated cancer-specific questionnaire addressing issues applicable to patients of different tumor types. It consists of a global health status (GHS) scale, 5 functioning scales (physical, role, cognitive, emotional and social), 3 symptom scales (fatigue, pain and nausea), and 6 single items (dyspnea, loss of appetite, insomnia, constipation, diarrhea and financial difficulties) [16]. The QLQ-OG25 is a disease-specific questionnaire for patients with esophagogastric cancer supplementing the QLQ-C30. This module assesses 1 functioning scale (body image) and 15 disease-specific symptoms associated with esophagogastric cancer, such as dysphagia, odynophagia, and reflux [17]. Maximum score for both the QLQ-C30 and QLQ-OG25 is 100. Scores are transformed to a 0–100 scale, where higher functioning scores indicate better HRQoL, and higher symptom scores represent more severe symptoms.

### Statistical analyses

Outcomes of this study were all the scales and single items of the QLQ-C30 and QLQ-OG25 questionnaires. Categorical variables were displayed in numbers and percentages and continuous variables in median with range. Missing data were managed according to the EORTC scoring manual [18].

Mean scores for each outcome were plotted and compared between baseline and subsequent time points. Multivariable linear mixed models were used to model all HRQoL outcomes over time. Variables in the model included all timepoints, World Health Organization (WHO) performance status at time of diagnosis, number of comorbidities at time of diagnosis, clinical tumor stage, and age at diagnosis. In case predictors in the model were statistically significant, an interaction term was included that modeled the interaction between the significant covariate and timepoints and as such tested for differential effects over time. For example, if performance status was a significant predictor in the model, the model tested if and how the course of HRQoL over time differed across levels of performance status.

All significance tests were considered statistically significant when *p* < 0.01, to correct for multiple testing. Significant mean changes of QLQ-C30 outcomes from baseline were considered clinically relevant if they exceeded previously defined minimally important differences [19]. Differences in QLQ-OG25 items were interpreted as small (5 to 10 points), medium (10 to 20 points) or large (> 20 points) [20]. All analyses were performed in RStudio (R version 4.0.3) and in SAS (version 9.4).

## Results

Data from 385 EC patients who each received at least one cycle of carboplatin and paclitaxel with a total radiotherapy dose of ≥ 42.0 Gy or a treatment duration of ≥ 30 days were extracted from the NCR. Of these, 67 were excluded owing to no available questionnaires returned within predefined time windows (*n *= 47) or treatment with chemotherapy other than carboplatin and paclitaxel (*n* = 20). Out of 318 included patients alive at baseline, 223 (70.1%) returned a baseline questionnaire before or within 7 days of starting treatment. Ninety-five patients (29.9%) did not complete a baseline questionnaire but did return questionnaires at later time points. A total of 718 questionnaires were answered across all prespecified timepoints. Eighty out of 307 alive patients at 3 months returned a questionnaire (26.1%), 88 out of 293 alive patients (30.0%) at 6 months, 85 out of 270 at 9 months (31.5%), 66 out of 252 at 12 months (26.2%), 105 out of 222 at 18 months (47.3%), and 71 out of 195 at 2 years (36.4%). A total of 185 (58.2%) patients died at the two years’ time point. Cumulative mortality was 11 at 3 months, 25 at 6 months, 48 at 9 months, 66 at 12 months, 96 at 18 months, and 123 at 2 years. The number of missing data per timepoint and item scale is provided in Supplementary Table S1, and ranges from 0% to 5.9%. The symptom score hair loss has a high amount of missing data across all timepoints (*n* = 457), which is due to the official EORTC questionnaire asking patients to only answer the question if a patient lost any hair.

### Patient characteristics and baseline HRQoL

The demographic and tumor characteristics of the 318 included patients are presented in Table 1. The median age was 73 (range 47–88) and patients were predominantly male (72.0%). Histological subtypes were adenocarcinoma (50.3%), squamous cell carcinoma (49.1%), mixed adenoneuroendocrine carcinoma (0.3%) and not otherwise specified (NOS) (0.3%). The majority of patients had a WHO performance status of 0 (40.3%) or 1 (42.1%) at diagnosis. The mean GHS at baseline was 70.6 (95% CI 68.1–73.1). At baseline, highest mean symptom scores were observed for anxiety (50.7), eating restrictions (35.9), dysphagia (28.7), odynophagia (26.0) and fatigue (25.7) (Table S1).

**Table 1:** Patient, tumor and treatment characteristics

Baseline characteristics		n	%
		318	100
Age (years) (median, range)		73 (47–88)	
Gender	Male	229	72.0
	Female	89	28.0
WHO performance status	0	128	40.3
	1	134	42.1
	2	34	10.7
	3	3	0.9
	Unknown	19	6.0
Comorbidities	0	123	38.7
	1	80	25.2
	≥ 2	104	32.7
	Unknown	11	3.5
Tumor location in esophagus	Upper third	53	16.7
	Middle third	74	23.3
	Lower third	165	51.9
	GEJ	18	5.7
	Other	8	2.5
Histological subtype	Adenocarcinoma	160	50.3
	Squamous cell carcinoma	156	49.1
	Other^a^	2	0.6
Clinical T stage	0	0	0.0
	1	4	1.3
	2	77	24.2
	3	196	61.6
	4	28	8.8
	X	13	4.1
Clinical N stage	0	131	41.2
	1	103	32.4
	2	69	21.7
	3	11	3.5
	X	4	1.3
Clinical M stage	0	286	89.9
	1	32	10.1
Cycles of chemotherapy received	≤ 4	32	10.1
	5	71	22.3
	6	209	65.7
	Unknown	6	1.9
Dose radiotherapy received (Gy) (median, range)		50.4 (43.2–61.6)	

^a^Mixed adenoendocrine carcinom (*n* = 1) and not otherwise specified (*n* = 1). *GEJ* = gastro-esophageal junction, *WHO* = World Health Organization

### Global health score and functioning scales

**Fig. 1:**
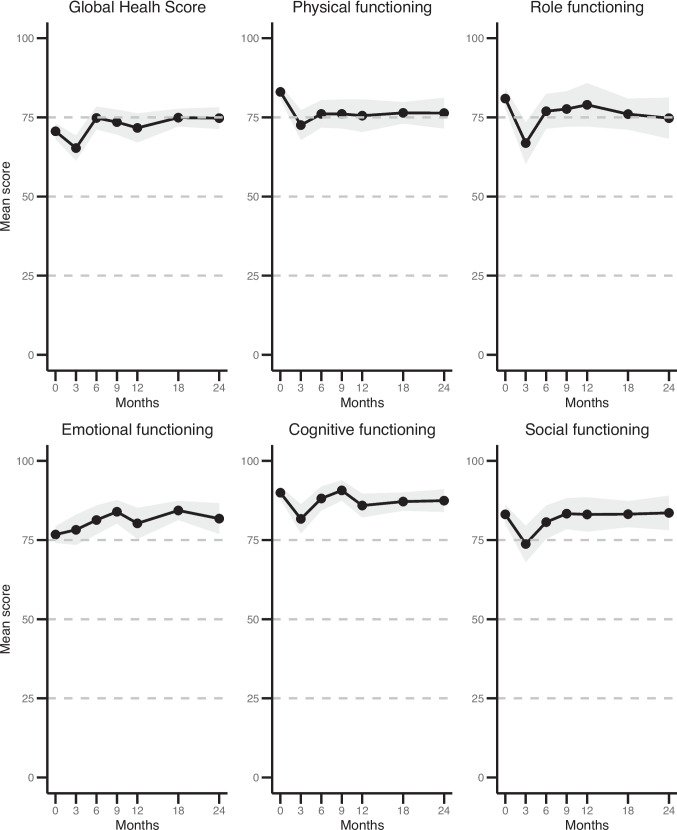
Mean scores of QIQ-C30 functioning scales

**Fig. 2:**
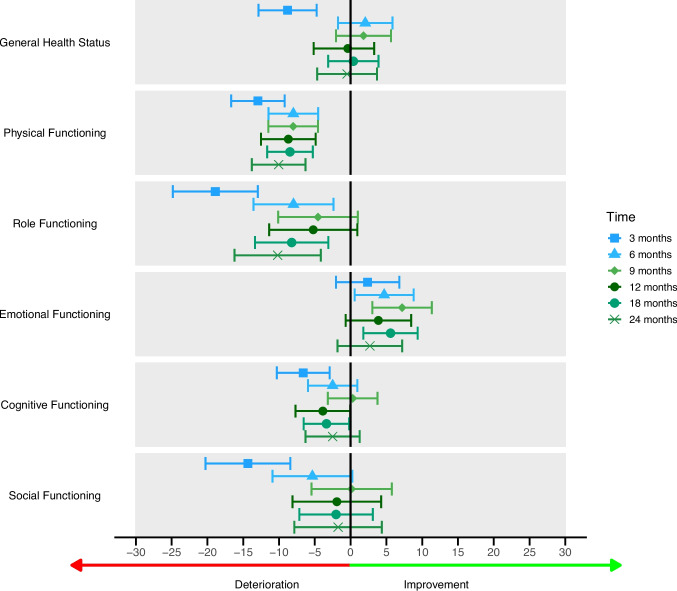
QLQ-C30 functioning scales: mean score changes compared to baseline

All changes in mean scores across the pre-specified timepoints of QLQ-C30 functioning scales are shown in Fig. 1. Figure 2 illustrates the extent of improvement or deterioration of the functioning scales at the pre-specified timepoints in comparison to baseline.

Mean scores and 95% CI for the EORTC QLQ-C30 functioning scales at baseline, 3 months, 6 months, 9 months, 12 months, 18 months and 24 months follow-up. The size of the data point indicates the number of returned questionnaires at that time point. The 95% CI is shown in grey. The number of returned questionnaires was 223 out of 318 alive patients (70.1%) at baseline, 80 out of 307 (26.1%) at 3 months, 88 (30.0%) at 6 months, 85 (31.5%) at 9 months, 66 (26.2%) at 12 months, 105 (47.3%) at 18 months, and 71 (36.4%) at 2 years. The number of missing data per timepoint and item scale is provided in Supplementary Table S1, and ranges from 0% to 5.9%.

Mean score changes and 95% CI compared to baseline for the EORTC QLQ-C30 functioning scales and Global Health Score.

GHS significantly declined at 3 months post-dCRT compared to baseline, but recovered to baseline at 6 months and remained stable over time (Table S2). Patients with performance status ≥ 1 scored on average 8.9 points lower compared to patients with performance status 0 (*p* < 0.001), but no differential effect over time was observed. Each additional year of age at diagnosis corresponded to 0.42 point increase in GHS score (*p* = 0.0002), but without a differential effect over time.

Physical functioning significantly deteriorated over all timepoints. The largest clinically relevant deterioration was observed between baseline and 3 months (mean difference [MD] = −13.0 [95% CI: −16.68—−9.22]; *p *< 0.001). Patients with a performance status ≥ 1 scored on average 11.2 points lower (*p* < 0.001), but again no differences over time were observed. Role functioning showed a significant deterioration compared to baseline at 3, 6, 18 and 24 months (largest MD at 3 months: −18.9 [−24.8−13.0]; *p *< 0.001). Social functioning and cognitive functioning only showed deterioration at 3 months in comparison to baseline (MD SF: −14.4 [−20.3−8.4], *p* < 0.001; MD CF: −6.6 [−10.3−2.9], *p* = 0.0005). While no differences over time were found, generally patients with a performance status ≥ 1 scored lower on role functioning, social functioning and cognitive functioning, with the largest effect on role functioning (MD: −12.4 [−17.8−6.9]; p < 0.001). Each additional year of age at diagnosis corresponded to an increase in mean scores for role functioning and social functioning. Contrary to previous outcomes, emotional functioning seemed to improve over time but was only clinically relevant and significantly better at 9 months compared to baseline (MD: 7.2 [3.0–11.3]; *p *= 0.0007). Patients with a performance status ≥ 1 scored lower on emotional functioning (MD: −7.7 [−11.8−3.5]; *p* = 0.0003), but with no differential effects over time.

Body image worsened at 3 and 6 months compared to baseline (largest MD at 3 months: −10.8 [−16.4–4.7]; *p* = 0.0005), after which no significant change from baseline was observed. Patients with a performance status ≥ 1 reported more problems with body image (MD: −8.4 [−14.0−2.8]; *p* = 0.003).

No clinically relevant and significant differences were found in the comparison of functioning scores between patients with one or more pre-existing comorbidities and patients without comorbidities, nor between patients with tumor stage 1–2 and stage 3–4.

### Symptom scores

**Fig. 3:**
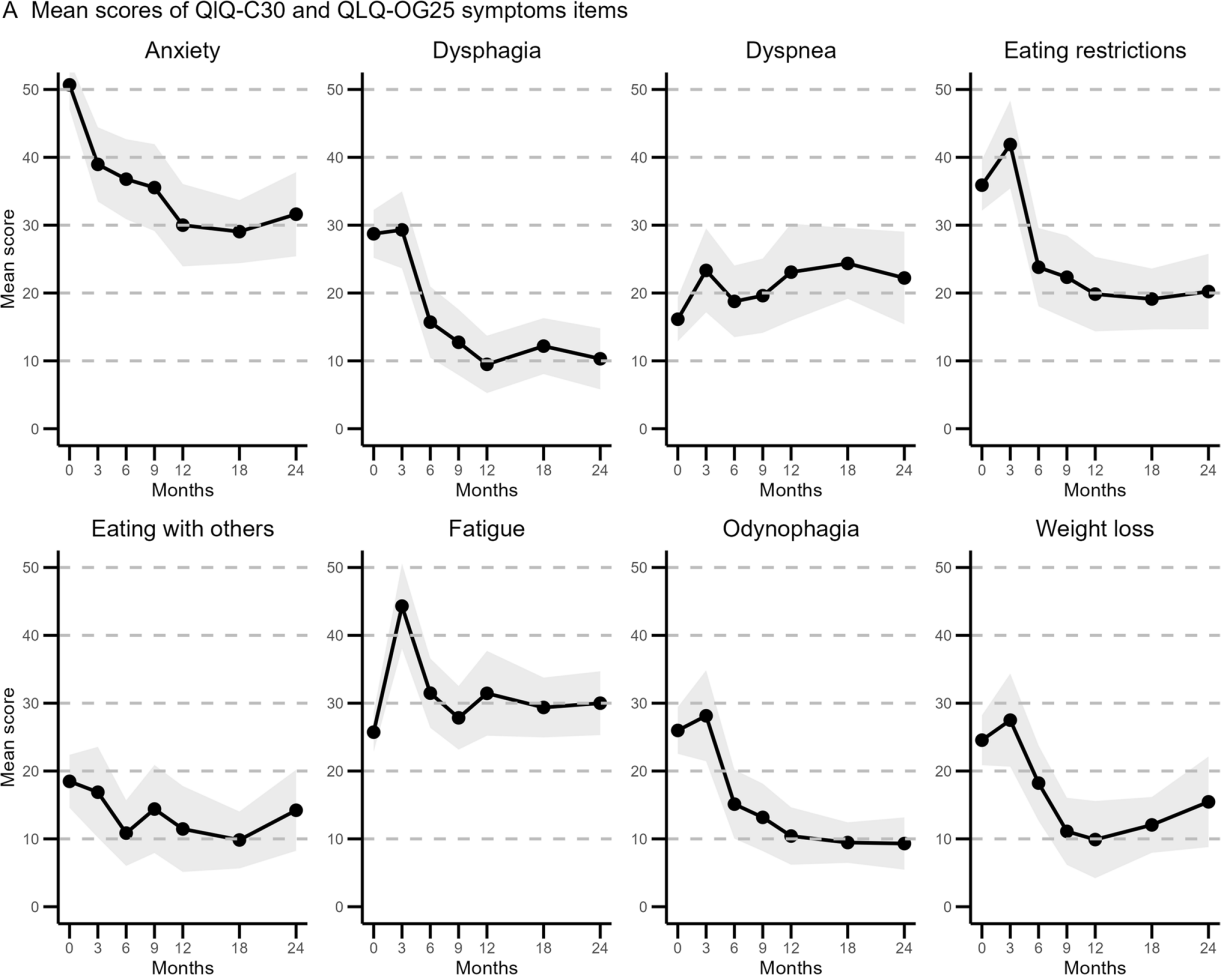

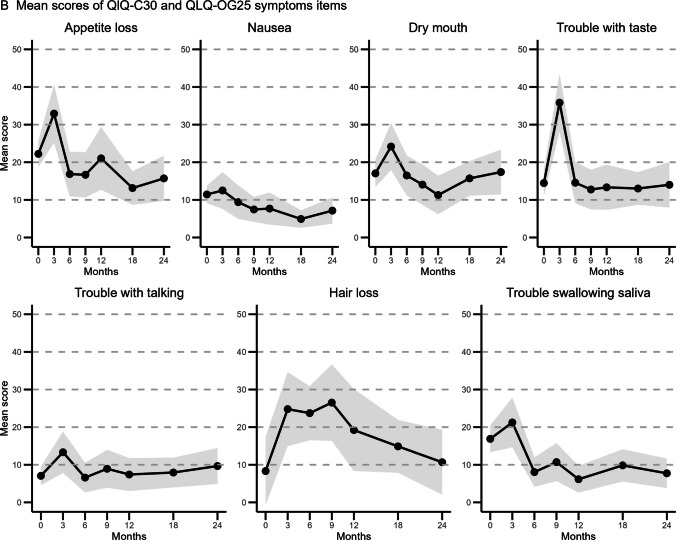
Mean scores and 95% CI for selected EORTC QLQ-C30 and EORTC-OG25 symptom items at baseline, 3 months, 6 months, 9 months, 12 months, 18 months and 24 months follow-up. The size of the data point indicates the number of returned questionnaires at that time point. The 95% CI is shown in grey. The number of returned questionnaires was 223 out of 318 alive patients (70.1%) at baseline, 80 out of 307 (26.1%) at 3 months, 88 (30.0%) at 6 months, 85 (31.5%) at 9 months, 66 (26.2%) at 12 months, 105 (47.3%) at 18 months, and 71 (36.4%) at 2 years. The number of missing data per timepoint and item scale is provided in Supplementary Table S1, and ranges from 0% to 5.9%

**Fig. 4:**
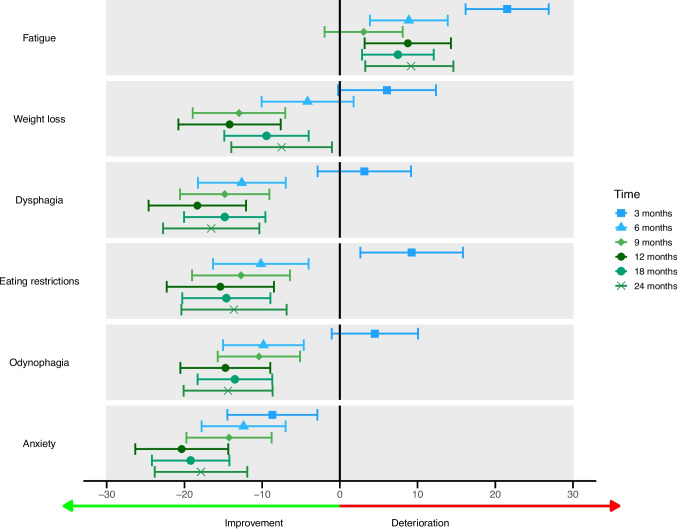
Symptom scores: mean score changes to compared to baseline.

All changes in mean scores across the pre-specified timepoints of QLQ-C30 and QLQ-OG25 symptom scales are shown in Fig. 3. Figure 4 illustrates the extent of improvement or deterioration of the functioning scales at the pre-specified timepoints in comparison to baseline. The symptom scale anxiety showed significant and clinically relevant improvement across all timepoints, with the largest mean difference between baseline and 12 months (MD: −20.4 [−26.3−14.2]; *p* < 0.0001). Dysphagia and odynophagia scores improved after 6 months across all timepoints. Eating restrictions scores first increased at 3 months compared to baseline, but decreased across all other timepoints. Five other symptom scales only showed improvement at specific timepoints; trouble with swallowing (at 6 and 12 months), weight loss (at 9, 12 and 18 months), nausea and vomiting (at 18 months), and eating in front of others (at 18 months). The symptom scales choking when swallowing and coughing showed no significant differences from baseline at any timepoint or for any variables included in the mixed model.

Mean score changes and 95% CI compared to baseline for selected EORTC QLQ-C30 and EORTC-OG25 symptom items.

In contrast, fatigue and dyspnea were found to worsen compared to baseline at multiple timepoints. Fatigue showed the largest clinically relevant difference (MD at 3 months: 21.5 [16.2–26.9]; *p* < 0.0001). Patients with > 1 pre-existing comorbidities and a performance status ≥ 1 reported higher dyspnea scores (respectfully, MD: 13.6 [6.3—20.9]; *p* = 0.0003, and MD 9.3 [3.6—15.0]; *p* = 0.0016). Mean scores for appetite loss, dry mouth, trouble with taste, and trouble with talking only increased at 3 months compared to baseline. Trouble with taste showed the largest mean difference (MD at 3 months: 25.4 [18.9–31.9]; *p* < 0.0001). Patients reported more hair loss problems at 3 and 9 months compared to baseline.

Each additional year of age was associated with a decrease in mean symptom scores for anxiety, dysphagia, odynophagia, eating restrictions, weight loss, eating in front of others, financial difficulty, pain, diarrhea and reflux. For odynophagia, the differential effect of older age was significant at 3 months compared to baseline (MD: − 0.9 per year of age [*p* = 0.009]). No age-by-time interactions were observed at other time points or for other symptom scales.

Patients with a performance status ≥ 1 reported significantly more severe symptoms for fatigue, dyspnea, dry mouth, trouble swallowing, dysphagia, eating restrictions, odynophagia, constipation, nausea and vomiting, insomnia, and esophagogastric pain. A differential effect for performance status was found for odynophagia at 12 months and for dyspnea at 3 months, which actually showed an improvement of symptoms compared to baseline (respectfully, MD: −16.1 [−27.7−4.6]; *p* = 0.006, and MD: −17.2 [29.0−5.4]; *p* = 0.0045).

## Discussion

This study aimed to investigate the course of HRQoL during and after definitive chemoradiotherapy with carboplatin and paclitaxel among patients diagnosed with EC. Consistent with previous reports [21], our findings indicate impairments in disease-specific symptoms, such as eating restrictions, odynophagia, and dysphagia at baseline. Compared to the general age-matched Dutch population, patients reported lower GHS prior to treatment (mean score of 78.6 [for Dutch patients 60–69 years of age] and 83.1 [70 + years of age] vs 70.6 in our study, respectively) [22].

In general, GHS declined at 3 months after dCRT compared to baseline. Importantly, and in line with previous studies [23–30], mean GHS scores remained stable compared to baseline from 6 months onwards. We also observed a clinically relevant deterioration at 3 months, which was on average 6.5 weeks after completion of treatment, of physical functioning, role functioning, cognitive functioning, social functioning, and the functioning scale body image. Social and cognitive functioning reached baseline levels around 6 months, and body image reached baseline levels around 9 months. Physical and role functioning did not return to baseline levels after 2 years.

Remarkably, a clinically relevant improvement was observed in emotional functioning and symptoms of anxiety, dysphagia, odynophagia, and eating restrictions, with these improvements persisting at 18 and/or 24 months post-treatment. These sustained improvements suggest a lasting benefit of definitive chemoradiotherapy on disease-specific symptoms. This is in line with a systematic review and meta-analysis reporting improvement in dysphagia compared to baseline post-definitive chemoradiation [13]. In contrast, treatment did not appear to have a significant impact on other symptoms such as esophagogastric pain, choking when swallowing, and pain, nor did it have a significant long-term effect on trouble talking, dry mouth, trouble swallowing saliva, or trouble with taste. It should be noted that many patients reported only mild complaints for several of these HRQoL items at baseline, leaving limited room for measurable improvement. Mean scores for appetite loss, dry mouth, trouble with taste, and trouble with talking only increased at 3 months compared to baseline, likely reflecting early radiation toxicity. These effects appear reversible, explaining return to baseline. Further improvement was limited because patients initially reported only mild complaints in this domain. Taken together, our results indicate that definitive chemoradiation alleviates the disease-specific symptoms that are most severe at baseline.

To the best of our knowledge, there are no previous studies that have reported improvements in emotional functioning after definitive chemoradiotherapy for EC. However, it has been observed that anxiety levels decrease after esophagectomy, especially in combination with neoadjuvant treatment [31]. Such a reduction in anxiety symptoms may be attributed to patients having sufficient time to adapt to their cancer diagnosis and regain a sense of control through treatment and the possibility of cure [32]. Consequently, the alleviation of anxiety symptoms may potentially lead to an improvement in overall emotional functioning.

Additionally, patients reported persistently clinically relevant higher levels of fatigue and dyspnea. The latter aligns with findings from the previously mentioned systematic review and meta-analysis [13]. The observed increase in dyspnea from baseline may be attributed to pulmonary toxicity caused by radiotherapy, which can be categorized into early and late phases. Early radiation-induced lung injury typically occurs within hours to days post-radiotherapy and may present as radiation pneumonitis, a condition that can be reversible. In contrast, late toxicity develops months to years post-radiotherapy and often presents as irreversible pulmonary fibrosis, which is associated with persistent dyspnea [33]. The increase in dyspnea observed at 3 months likely reflects this early phase of toxicity. Subsequently, some patients may recover from early lung injury, while others progress to permanent damage, beginning around 9 months and corresponding to the fibrotic phase.

It has previously been suggested that the incidence of pulmonary toxicity and dyspnea following radiotherapy may be more prevalent in patients with pre-existent respiratory conditions [25]. Our findings support this suggestion, as patients with pre-existent comorbidities like chronic obstructive pulmonary disease (COPD) had significantly higher dyspnea scores than those without (*n* = 54 out of 307 (excluding missing data); *n* = 35 out of 78 in category > 1 comorbidity). Based on these data, we recommend close monitoring of dyspnea symptoms in patients with pre-existing respiratory conditions to timely recognize possible late toxicity in these patients. Moreover, proton therapy may be considered, since this treatment modality is associated with a lower dose to the lungs and could therefore reduce radiation pneumonitis [34].

To our knowledge, no previous study has examined the HRQoL after definitive chemoradiotherapy regarding tumor stage and performance status. In our study, patients with poorer performance status frequently reported worse HRQoL. However, our results did not demonstrate significant associations between tumor stage and HRQoL scores. Interestingly, each additional year of age at diagnosis was frequently associated with better HRQoL scores compared to baseline. This could possibly be due to differences in health expectations and baseline activity levels between older and younger patients.

To address the persisting problems with physical functioning and the association of poorer performance status with worse HRQoL, patients could perhaps benefit from adequate supportive care during and after treatment. For example, a systematic review of 23 studies investigating physical activity after neoadjuvant chemoradiotherapy in EC patients found that those who participated in a preoperative exercise program experienced a smaller decrease in exercise ability compared to those who did not [35].

Engaging patients in a comprehensive discussion regarding the trade-offs between potential benefits, such as improved chances of survival, and the associated risks, such as potential impairments in HRQoL, is a crucial component of the shared decision-making process in cancer treatment [36]. Although many clinical trials primarily focus on survival outcomes, relatively few studies extensively report the impact of treatment on HRQoL. In our study, we observed that GHS remained stable after 6 months following treatment. However, using only GHS as a measure for HRQoL is not advisable as improvements in certain domains may balance out against deteriorations in other domains, or patients may adapt to a new situation. Indeed, in this study we observed clinically relevant improvements and deteriorations in different HRQoL domains. These findings can provide valuable insights to address concerns regarding the impact of definitive chemoradiotherapy on HRQoL within the context of shared decision-making.

To our knowledge, this is the first multicenter, real-world cohort study investigating HRQoL in patients with EC receiving definitive chemoradiotherapy. The multicenter design of our study enhances its generalizability by reducing the risk of selection bias commonly associated with single-center studies. The population included in the POCOP study has been shown to be representative for EC patients in the general Dutch population undergoing various treatments, such as definitive chemoradiotherapy [37].

This study has several limitations. Since this is an observational cohort study, it is uncertain to what extent the observed differences can be ascribed to chemoradiotherapy. Additionally, since a selective set of variables are recorded in the NCR, we were unable to correct for all relevant clinical variables that might influence treatment toxicity. For example, novel radiation techniques might reduce toxicity and subsequently affect HRQoL. Additionally, disease recurrence as well as possible subsequent treatments, such as salvage resections, were unknown and could have influenced the results. Furthermore, the results could have been influenced by a decline in response rate during follow-up, as patients with worse HRQoL may have stopped returning questionnaires, or patients may have died during follow-up. Therefore, it is important to note that the long-term results of this study reflect a specific subgroup of patients who are still alive at 2 years. Finally, the sample size of this study is relatively small, and multiple testing was necessary to be able to describe the course of all aspects of HRQoL over time. To account for this, differences were interpreted based on both statistical significance and clinical relevance.

Strengths of this study include the use of standardized, validated HRQoL questionnaires at set time points and its real-world heterogeneous population – including patients who are medically unfit for surgery, patients with inoperable tumors and patients who opt for non-surgical treatment. Future research into this topic could investigate whether HRQoL outcomes differ between these groups, as suggested by our data showing that worse performance status and comorbidities are significantly associated with worse HRQoL.

In conclusion, GHS remained stable after 6 months in patients receiving definitive chemoradiotherapy for EC. However, the burden of several disease-specific symptoms alleviated substantially over time, and emotional functioning, anxiety, and severely affected symptoms at baseline such as dysphagia, improved. Social and role functioning deteriorated but recovered afterward, whereas physical functioning, role functioning, fatigue and dyspnea deteriorated and did not recover.

## Supplementary information

Below is the link to the electronic supplementary material.

ESM 1 (XLSX 60.5 KB)

## Data Availability

Data is provided within the manuscript or supplementary information files. Access to unprocessed data can be requested through IKNL.
